# Type and dose of radiotherapy used for initial treatment of non-metastatic prostate cancer

**DOI:** 10.1186/1748-717X-9-47

**Published:** 2014-02-05

**Authors:** Dian Wang, Alex Ho, Ann S Hamilton, Xiao-Cheng Wu, Mary Lo, Steven Fleming, Michael Goodman, Trevor Thompson, Jean Owen

**Affiliations:** 1 Department of Radiation Oncology, Medical College of Wisconsin, Milwaukee, WI 53045, USA; 2 Clinical Research Center, American College of Radiology, Philadelphia, PA, USA; 3 Keck School of Medicine, University of Southern California, Los Angeles, CA, USA; 4 Louisiana Tumor Registry, LSU Health Science Center, New Orleans, LA, USA; 5 University of Kentucky College of Public Health, Lexington, KY, USA; 6 Department Epidemiology, Rollins School Public Health, Emory University, Atlanta, GA, USA; 7 Centers for Disease Control and Prevention, Atlanta, GA, USA

**Keywords:** Prostate cancer, Radiotherapy, Patterns of care study

## Abstract

**Background:**

We sought to describe patterns of initial radiotherapy among non-metastatic prostate cancer (PC) patients by recurrence risk groups.

**Methods:**

Medical records were abstracted for a sample of 9017 PC cases diagnosed in 2004 as a part of the Center for Disease Control and Prevention’s Prostate and Breast Patterns of Care Study in seven states. Non-metastatic PC cases are categorized as low-risk (LR), intermediate-risk (IR) or high-risk (HR) groups based on pretreatment PSA, tumor stage, and Gleason score per 2002 NCCN guidelines. Univariate and multivariate analyses were employed to determine factors associated with the type and dose of radiotherapy by the risk groups.

**Results:**

Of the 9,017 patients, 3153 who received definitive radiotherapy either alone or in combination with hormone therapy (HT) were selected for in-depth analysis. Multivariate models showed that LR patients were more likely to receive seed implant brachytherapy (BT) than those in higher risk groups. Those in the IR group were most likely to receive external beam radiotherapy (EBRT) combined with BT or high-dose radiotherapy. Use of HT in combination with radiotherapy was more common in the IR and HR groups than for LR patients. Intensity modulated radiation treatment (IMRT) was used to treat 32.6% of PC patients treated with EBRT, with the majority (60.6%) treated with high-dose radiotherapy.

**Conclusions:**

Radiotherapy types and dosage utilization varied by PC risk groups. Patients in IR were more likely than those in LR or HR to receive high-dose radiotherapy. IMRT was used in about one third of patients to deliver high-dose radiotherapy.

## Background

Definitive radiotherapy (RT), either alone or in combination with hormone therapy (HT), has been commonly used to treat patients with non-metastatic prostate cancer in accordance with the National Comprehensive Cancer Network (NCCN) guidelines [1]. However, multiple options for initial treatment including external beam radiation treatment (EBRT), brachytherapy, surgery or various combinations of these with or without HT exist for patients with similar risk factors. In addition, treatment choice is influenced by demographic and socioeconomic factors, medical comorbidities and concerns about the adverse effects of therapy [1]. Furthermore, new technologies and therapies have been introduced into clinical practice. There have been significant increases in the use of intensity modulated radiation treatment (IMRT), and HT combined with radiotherapy (RT) for non-metastatic PC. However, population-based information on radiotherapy types and dosages by NCCN recurrence risk groups is lacking in the literature. Because population-based cancer registries do not collect up-to-date radiotherapy data of these types and dosage, special data collection is required to conduct such analysis.

In response to suggestions by the Institute of Medicine [2], the National Program of Cancer Registries (NPCR) funded by the Center for Disease Control and Prevention (CDC) undertook a multi-state Patterns of Care study to evaluate the standard of practice for cancer treatment in a cross-section of the United States. A Prostate Cancer Data Quality and Patterns of Care Study (POC BP) in 1997 was previously reported [3]. Practice trends in PC were described based on individual risk factors such as prostate specific antigen (PSA), Gleason score and results (normal or abnormal) of digital rectal examination (not clinical T stage) [3]. The current study has expanded data routinely collected by the State cancer registry for PC patients diagnosed in 2004 in seven States (California, Georgia, Kentucky, Louisiana, Minnesota, North Carolina, and Wisconsin). We previously reported clinical and demographic factors associated with receipt of guideline concordant initial therapy for localized PC [4]. The current analysis evaluates the type and dose of radiotherapy used for initial treatment of localized PC having different risk estimates of recurrence as defined in the NCCN guidelines [5].

## Materials and methods

Prostate cancer patient records were re-abstracted from a stratified sample of incident microscopically-confirmed prostate cancer cases diagnosed in 2004 from seven states (i.e., California, Georgia, Kentucky, Louisiana, Minnesota, North Carolina, and Wisconsin) as part of the CDC’s POC-BP conducted in 2007-2009. Cancer registry data obtained by routine methods [6] were verified and supplemented by re-abstracting hospital records and by obtaining additional treatment information from outpatient facilities including physicians’ offices, ambulatory surgery centers, radiation facilities, and long-term care facilities. Further information was collected regarding the patient demographic characteristics, clinical features of the tumor, work-up details, and the first course of cancer-directed treatment (i.e. therapy regimen that was given at the time of the initial diagnosis, prior to disease recurrence or progression).

The sample methodology, the design of the POC-BP study, and an analysis of cancer registry data quality have been reported elsewhere [7,8]. Briefly, 11,679 PC cases were randomly selected across strata defined by race/ethnicity and state-specific factors, for example, Appalachian vs. non-Appalachian region, type of facility, or patient volume of the facility. Abstraction was completed for 77.2% (9,017) of the sampled cases. Of those, 350 patients with node positive (N1) or metastatic (M1) disease, 162 with insufficient information for NCCN recurrence risk group classification and 33 who were deceased within 6 months of diagnosis with no treatment was excluded from the analysis. A total of 3,153 of the remaining 8,472 patients who had RT as initial treatment were included in an in-depth analysis. The study was approved by institutional review boards at participating institutions, from 6 of the 7 participating states (one was exempted), and at CDC.

### Patient socio-demographic factors

Age at diagnosis, race and ethnicity, marital status, and health insurance coverage were abstracted from medical records. Other information pertaining to the patient’s census tract of residence was obtained from 2000 U.S. Census data. The census tract-specific indicators included urbanization (100% urban, 100% rural, urban/rural mix), proportion of individuals in the working class (<66% vs. 66%+), proportion of the population below the federal poverty level (<20% vs. 20%+), and proportion of persons (>25 years of age) without a high school education (<25% vs. 25%+).

### Definition of clinical recurrence risk groups

Clinical recurrence risk groups were defined according to the NCCN guidelines for Prostate Cancer (version 1.2002) that were in effect at the time these patients were originally diagnosed [5]. Briefly, parameters used to characterize the risk of recurrence includes pretreatment prostatic specific antigen (PSA), clinical T stage and Gleason score (GS) from needle biopsy samples. Patients in the low risk group (LR) had T1-T2a tumors, GS 2-6, and PSA < 10 ng/ml. The intermediate risk group (IR) include those with T2b-T2c tumors or GS = 7 or PSA 10-20 ng/ml, and the high/very high risk group (HR) had T3a-T4 tumors or GS 8-10 or PSA > 20 ng/ml. The American Joint Committee on Cancer Clinical Staging System 6th edition [9] was used to assign the clinical T stage.

### Definition of comorbidity

The ACE-27 (Adult Comorbidity Evaluation-27) was used to measure comorbidity burden because of its clinical relevance and sensitivity [7]. This 26-item chart-based comorbidity index was initially developed by Piccirillo and colleagues [10] and has been subsequently validated [11,12]. The index is based on 26 comorbid conditions, with three grades of decompensation (or severity) [12] that were present at or before the date of diagnosis. Therefore, complications related to cancer and/or its treatment were not scored as comorbidities. Overall the comorbidity severity score for the ACE-27 (Severe, Moderate, Mild, or None) used in this study was determined by the highest ranking single condition, except when two or more Grade 2 conditions occurred in different organ systems, in which case the overall comorbidity score was classified as Severe (Grade 3).

### Definition of initial treatment

Initial treatment is defined as treatment received within the first six months following pathological diagnosis of prostate adenocarcinoma. In this report, initial RT includes EBRT alone or seed implant brachytherapy (BT) alone or a combination of both with or without HT. High-dose-rate brachytherapy data was not abstracted in all seven states and was therefore excluded for this analysis. The total dose Gray (Gy) administered is abstracted from medical records for patients who completed definitive EBRT only, but the fractionation schedule was unknown. HT is defined as receiving any type of anti-androgen therapy.

### Statistical analysis

Data analyses are performed using statistical software SAS v.9.2, and SUDAAN v.10 that handles complex sample surveys and allows for weighted estimates. Categorical variables are presented as unweighted numbers (n_uw_), weighted numbers (n_w_) and percentages (%) of patients. Unadjusted associations of socio-demographic and tumor characteristics with treatment modality are examined by chi-square tests. Multivariable logistic regression models are constructed to investigate the association of initial RT patterns of PC care with the recurrence risk groups. Covariates included in the initial model are the seven registries, patient socio-demographics (age, race and ethnicity, marital status, education, employment, poverty level, urbanization, health insurance) and co-morbidity. Those covariates that do not show significant relationship in the initial model are excluded in the final reported model, except co-morbidity, since it is a clinically important factor that would determine a patient’s treatment. Odds ratios (OR) and 95% confidence intervals (95% CI) were calculated with results considered statistically significant at a two-sided alpha-error level of <0.05.

## Results

Among 8,472 patients with non-metastatic PC, 3,153 received definitive radiotherapy, either alone or in combination with HT. The characteristics of study participants are summarized in Table 1. The vast majority of patients receiving RT (85.3%) were 60 years of age or older. Non-Hispanic Whites accounted for approximately 70% of the cases followed by non-Hispanic Black (19.4%), Hispanic (5.6%) and others (2.6%). Of those receiving initial RT, 54.9% had EBRT alone, 26.1% received BT alone, 17.6% had EBRT combined with BT, and 1.4% had an unknown RT modality. Variations in the use of RT by modality are evident among the participating study sites (e.g. combination therapy was more likely to occur in Registry B, whereas EBRT alone was the most common treatment in Registry A). All socio-demographic characteristics evaluated, except marital status and urbanization, were associated with variations of initial RT modalities (p < 0.0001). Men aged 70+ years were more likely than younger men to receive EBRT alone. Non-Hispanic whites had the highest proportion receiving BT alone compared with other racial/ethnic groups. Men living in high poverty areas were more likely than their counterparts to receive EBRT alone.

**Table 1:** Patient and tumor characteristics by type of initial radiation treatment

**Characteristics**	Total (%)^*^	**Patients (n)**^**†**^	**BT alone (%)**^**‡**^	**EBRT alone (%)**^**‡**^	**EBRT+BT (%)**^**‡**^	**Others**^**§ **^**(%)**^**‡**^
Total	100.0	8946 (3153)	26.1	54.9	17.6	1.4
States (p < 0.0001)^¶^						
A	22.6	2025 (502)	20.9	67.1	8.3	3.7
B	22.7	2028 (906)	21.2	43.9	34.1	0.8
C	6.9	618 (174)	30.4	59.1	9.9	0.6
D	10.0	892 (586)	30.8	50.8	16.2	2.2
E	8.7	776 (251)	25.7	53.0	21.3	0.0
F	19.4	1739 (433)	26.7	57.4	15.9	0.0
G	9.7	868 (301)	40.3	49.8	8.1	1.8
Age (y) (p < 0.0001)^¶^						
20–59	14.7	1316 (499)	27.8	43.5	27.4	1.3
60–69	37.7	3375 (1251)	29.5	49.4	20.1	1.0
70+	47.6	4255 (1403)	22.8	62.7	12.7	1.8
Race (p < 0.0001)^¶^						
White, non-Hispanic	72.4	6478 (1805)	28.4	52.1	18.4	1.1
Black, non-Hispanic	19.4	1737 (1002)	20.1	61.0	18.1	0.8
Hispanic	5.6	497 (195)	19.7	65.4	7.0	7.9
Asians/others	2.6	234 (151)	18.8	63.1	16.1	2.0
Marital status (p = 0.2000)^¶^						
Single	22.6	1949 (730)	24.0	58.2	16.9	0.9
Married	77.4	6680 (2317)	27.0	53.2	18.3	1.5
Education^§^ (p < 0.0001)^¶^						
Not undereducated	64.5	5751 (1787)	29.0	51.0	18.7	1.3
Undereducated	35.5	3166 (1357)	21.0	61.7	15.6	1.7
Working class (p = 0.0075)^¶^						
Not working class	43.7	3893 (1203)	28.2	50.5	19.8	1.5
Working class	56.3	5025 (1941)	24.5	58.2	15.9	1.4
Urbanization (p = 0.3671)^¶^						
Urban	48.5	4324 (1468)	26.4	54.7	17.2	1.7
Rural	15.4	1372 (525)	23.6	59.7	15.8	0.9
Urban-Rural mix	36.1	3221 (1151)	26.8	52.9	18.9	1.4
Poverty level (p = 0.0013)^¶^						
Not in poverty level	81.1	7233 (2317)	27.1	53.2	18.4	1.3
In poverty level	18.9	1685 (827)	21.7	61.9	14.4	2.0
Health insurance (p = 0.0010)^¶^						
Not insured	1.3	111 (50)	11.8	71.2	12.3	4.7
Public coverage	64.2	5535 (2009)	25.1	57.6	16.4	0.9
Private coverage	30.8	2660 (857)	27.8	48.4	21.5	2.3
Insurance, NOS	3.7	319 (131)	27.0	46.9	23.9	2.2
Piccirillo comorbidity score (p = 0.2893)^¶^						
None	28.8	2515 (860)	29.0	51.6	17.7	1.7
Mild	55.2	4816 (1720)	26.5	53.7	18.6	1.2
Moderate	12.2	1060 (390)	21.2	62.1	15.6	1.1
Severe	3.8	327 (111)	22.1	56.8	18.9	2.2
PSA (ng/ml) (p < 0.0001)^¶^						
<10	72.1	6360 (2199)	32.7	47.9	18.3	1.1
10–20	18.4	1620 (574)	10.2	69.8	18.0	2.0
>20	9.5	841 (329)	5.8	78.3	13.9	2.0
TNM Clinical T stage (p < 0.0001)^¶^						
Tx-T0	0.5	49 (18)	9.2	84.8	3.7	2.3
T1	64.8	5793 (2064)	31.3	51.5	16.2	1.0
T2	31.7	2832 (978)	18.2	58.9	21.0	1.9
T3-T4	3.0	268 (92)	0.0	80.1	16.0	3.9
Gleason score (p < 0.0001)^¶^						
2–6	56.6	5020 (1732)	37.5	46.4	14.7	1.4
7	31.9	2836 (1020)	13.0	62.8	22.9	1.3
8-10	11.5	1023 (375)	3.9	75.4	18.6	2.1
Recurrence risk group (p < 0.0001)^¶^						
LR	41.2	3684 (1257)	45.2	39.9	13.8	1.1
IR	40.1	3583 (1261)	16.1	60.0	22.5	1.4
HR	18.8	1680 (635)	5.2	76.8	15.7	2.3

*BT*, brachytherapy; *EBRT*, external beam radiation therapy; *LR*, low risk group; *IR*, intermediate risk group; *HR*, high/very high risk group.

(*) Column percentages based on weighted number of patients.

(†) Weighted number of patients, with unweighted number in parentheses.

(‡) Row percentages based on weighted number of patients.

(§) Included those with no RT, had RT but with unknown modality, and unknown if they had RT.

(¶) p-values (Chi-square test to measure of association between RT treatment and each of socio-demographic/clinical variables).

There was no significant relationship (p = 0.2893) between comorbidity severity and RT modality (Table 1). All tumor characteristics, however, are significantly associated with the type of RT received (p < 0.0001). Patients with PSA <10 ng/ml were more likely to receive BT only, whereas those with PSA >20 ng/ml were more likely to receive EBRT. Similarly, those with lower T stage (T1-2) had a higher likelihood of receiving BT only. In contrast, over 80% of those with T3-T4 disease received EBRT. More patients with T2 tumors received EBRT+BT than those with higher or lower T stages, and similarly, EBRT+BT was more commonly used for patients with a GS 7 than among those with higher or lower GS.

Among patients receiving initial RT, 41.2%, 40.1%, and 18.8% were in the LR, IR and HR groups, respectively. LR patients were more likely to receive BT, while EBRT was more likely to occur among those in the IR and HR groups. Receipt of EBRT+BT, suggestive of high-dose RT, was most common in the IR group.

Detailed information on receipt of EBRT, EBRT+BT or BT with/without HT by characteristics of patients is summarized in Table 2. Tumor characteristics such as T stage and GS are significant determinants of RT type either alone or in combination with HT. Men receiving RT (any type)+HT were more likely to have higher PSA levels, T stage, and GS than those who received RT alone.

**Table 2:** Comorbidity and tumor characteristics of patient by initial radiation treatment modality during the 6 months after diagnosis

**Comorbidity/tumor characteristics**	EBRT alone (%)^*^	EBRT+HT (%)^*^	EBRT+BT (%)^*^	EBRT+BT+HT (%)^*^	BT alone (%)^*^	BT+HT (%)^*^	RT (Type unknown)±HT (%)^*^
Total	24.5	30.4	10.5	7.1	17.9	8.2	1.4
Piccirillo comorbidity score (n_w_ = 8718, p = 0.5183)^†^							
None	28.5	26.6	31.0	24.6	33.9	26.7	35.3
Mild	54.4	54.9	55.0	60.2	52.9	60.9	49.3
Moderate	12.9	14.8	10.5	10.6	10.1	9.1	9.6
Severe	4.2	3.7	3.5	4.6	3.1	3.3	5.8
PSA (ng/ml) (n_w_ = 8822, p < 0.0001)^†^							
<10	76.3	52.2	83.0	60.9	92.7	86.3	59.0
10–20	20.5	25.7	13.6	25.8	5.9	10.1	27.0
>20	3.2	22.1	3.4	13.3	1.4	3.6	14.0
Clinical T stage (n_w_ = 8941, p < 0.0001)^†^							
Tx-T0	0.8	0.9	0.0	0.3	0.1	0.4	0.9
T1	70.5	53.0	66.3	49.4	77.0	79.2	46.7
T2	27.4	39.2	32.4	45.5	22.9	20.4	44.1
T3-T4	1.3	6.9	1.3	4.8	0.0	0.0	8.3
Gleason score (n_w_ = 8879, p < 0.0001)^†^							
2–6	66.7	32.3	56.4	32.6	84.6	76.7	55.1
7	29.5	42.2	38.0	46.0	14.0	20.7	27.9
8–10	3.8	25.5	5.6	21.4	1.4	2.6	17.0
Recurrence risk group (n_w_ = 8946, p < 0.0001)^†^							
LR	46.7	16.5	43.9	15.0	75.4	62.7	31.2
IR	46.1	41.8	47.3	56.7	21.8	31.4	38.8
HR	7.2	41.7	8.8	28.3	2.8	5.9	30.0

*LR*, low risk group; *IR*, intermediate risk group; *HR*, high/very high risk group; *BT*, brachytherapy; *EBRT*, external beam radiotherapy; *HT*, hormone therapy.

*RT*, radiotherapy.

(*) Column percentages based on weighted number of patients, except the first row (Total) which was a row percentage.

(†) p-values (Chi-square test to measure of association between each of comorbidity/tumor characteristics variables and initial treatment modality).

Significant differences are also observed across the recurrence risk groups in the percent of patients receiving RT alone versus RT+HT (Table 2). Patients receiving RT (any type)+HT were more likely to be in the HR group than those who received RT alone. A similar trend was also observed in the IR group. By contrast, patients receiving RT alone were more likely to be in the LR group than those who received RT+HT.

### Results of multivariate logistic models

**Figure 1:**
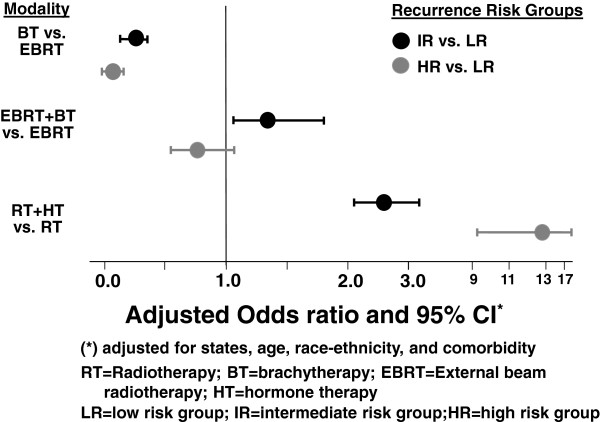
Multivariate logistic models.

Multivariate logistic models were constructed to measure the relationship between RT modality and recurrence risk groups, adjusting for comorbidity and sociodemographic factors (Figure 1). Results of this analysis show that compared with LR group, patients in higher risk groups were less likely to receive BT alone vs. EBRT (IR: OR = 0.26, 95% CI = 0.20-0.33; HR: OR = 0.06, 95% CI = 0.04-0.10). However, those with IR were more likely than those with LR to receive EBRT + BT (OR = 1.35, 95% CI = 1.01-1.80). Patients in IR (OR = 2.61, 95% CI = 2.10-3.24) or HR (OR = 12.57, 95% CI = 9.26-17.06) were more likely to receive RT plus HT compared with patients in LR.

The total dose of EBRT received (<70 Gy, 70-74 Gy, or ≥75 Gy) by sociodemographic, clinical and tumor characteristics, and recurrence risk group are shown in Table 3. Over half (53%) received less than 70 Gy and a quarter received 70-74 Gy while only 22% received a high dose (≥75 Gy). Of those receiving the high dose, 60.6% were treated with IMRT compared with 22.6% of those treated to 70-74 Gy and 26.7% of those treated with low dose (<70 Gy) (Table 4).

**Table 3:** Patient and tumor characteristics by a total dose of external beam radiation therapy (EBRT) alone

	**<70 Gy**		**70–74 Gy**		**75+ Gy**	
**Characteristics**	(%)^*^	**Patients (n)**^**†**^	(%)^*^	**Patients (n)**^**†**^	(%)^*^	**Patients (n)**^**†**^
Total^‡^, n = 4611 (1636)	53.0	2444 (920)	25.0	1152 (382)	22.0	1015 (334)
States (p < 0.0001)^§^						
A	32.2	369 (100)	41.5	476 (125)	26.3	302 (69)
B	74.3	645 (293)	9.8	85 (39)	15.9	138 (69)
C	58.5	204 (57)	27.5	96 (28)	14.0	49 (13)
D	57.7	253 (166)	26.7	117 (78)	15.6	68 (45)
E	46.2	188 (74)	13.0	53 (19)	40.8	167 (48)
F	59.5	584 (157)	22.4	221 (52)	18.1	177 (50)
G	48.0	201 (73)	24.8	104 (41)	27.2	114 (40)
Age (y) (p = 0.0156)^§^						
20–59	64.6	345 (131)	15.2	81 (33)	20.2	108 (37)
60–69	53.5	837 (338)	25.5	400 (141)	21.0	330 (127)
70+	50.3	1262 (451)	26.7	671 (208)	23.0	577 (170)
Race (p < 0.0001)^§^						
White, non-hispanic (NH)	53.5	1706 (508)	24.4	777 (190)	22.1	703 (183)
Black, NH	59.8	603 (332)	21.2	213 (123)	19.0	191 (106)
Hispanic	32.1	93 (52)	38.8	112 (38)	29.1	84 (23)
Asians/others	32.9	42 (28)	38.5	50 (31)	28.6	37 (22)
Marital status (p = 0.8262)^§^						
Single	55.0	586 (238)	23.8	253 (92)	21.2	225 (84)
Married	53.2	1773 (649)	23.8	795 (263)	23.0	766 (243)
Education^¶^ (p = 0.0279)^§^						
Not undereducated	50.8	1417 (481)	24.6	685 (200)	24.6	688 (195)
Undereducated	56.3	1014 (435)	25.8	464 (181)	17.9	323 (138)
Working class (p = 0.0910)^§^						
Not working class	48.9	908 (312)	26.3	487 (133)	24.8	461 (127)
Working class	55.7	1522 (604)	24.2	662 (248)	20.1	550 (206)
Urbanization (p = 0.0293)^§^						
Urban	47.6	1023 (400)	28.3	608 (200)	24.1	519 (165)
Rural	57.4	454 (165)	22.7	179 (64)	19.9	157 (57)
Urban-Rural mix	57.8	954 (351)	21.9	362 (117)	20.3	335 (111)
Poverty level (p = 0.0255)^§^						
Not in poverty level	51.6	1870 (644)	24.9	904 (268)	23.5	852 (254)
In poverty level	58.1	561 (272)	25.4	245 (113)	16.5	159 (79)
Health insurance (p = 0.0036)^§^						
Not insured	58.7	46 (22)	22.7	18 (6)	18.6	14 (7)
Public coverage	56.9	1732 (664)	20.9	637 (229)	22.2	674 (232)
Private coverage	45.1	519 (179)	36.4	418 (117)	18.5	212 (65)
Insurance, NOS	61.6	87 (33)	16.5	23 (11)	21.9	31 (10)
Piccirillo comorbidity score (p = 0.0800)^§^						
None	50.5	613 (221)	25.5	310 (94)	24.0	291 (88)
Mild	52.2	1273 (492)	24.8	604 (213)	23.0	560 (188)
Moderate	60.3	378 (143)	19.1	120 (44)	20.6	129 (42)
Severe	65.1	119 (46)	23.4	43 (11)	11.5	21 (10)
PSA (ng/ml) (p = 0.0016)^§^						
<10	50.0	1446 (533)	27.3	787 (256)	22.7	656 (210)
10–20	53.7	556 (215)	21.2	220 (74)	25.1	259 (81)
>20	65.1	408 (158)	21.0	131 (49)	13.9	87 (40)
Clinical T stage (p = 0.1833)^§^						
Tx-T0	26.1	9 (4)	24.3	9 (4)	49.6	18 (5)
T1	52.1	1477 (569)	26.2	741 (252)	21.7	613 (197)
T2	53.7	825 (299)	24.4	376 (115)	21.9	336 (115)
T3-T4	64.4	133 (48)	12.8	26 (11)	22.8	47 (17)
Gleason score (p = 0.0007)^§^						
2–6	46.9	1033 (385)	30.5	670 (218)	22.6	498 (163)
7	57.6	958 (361)	20.5	341 (115)	21.9	365 (119)
8–10	61.4	441 (169)	17.8	128 (46)	20.8	150 (50)
Recurrence risk group (p = 0.0001)^§^						
LR	44.3	620 (229)	34.0	477 (155)	21.7	304 (101)
IR	53.8	1071 (401)	21.9	435 (143)	24.3	484 (150)
HR	61.8	753 (290)	19.7	241 (84)	18.5	226 (83)

*LR*, low risk group; *IR*, intermediate risk group; *HR*, high/very high risk group.

(*) Row percentages based on weighted number of patients.

(†) Weighted number of patients, with unweighted number in parentheses.

(‡) Excluded 101 patients (unweighted) who did not have information on EBRT dose.

(§) p-values (Chi-square test to measure of association between EBRT dose and each of socio-demographic/clinical variables).

**Table 4:** Relationship between total EBRT dose and EBRT modality

**RT modality (p < 0.0001)**	**<70 Gy**	**70–74 Gy**	**75+ Gy**
non-IMRT	69.8^*^	77.4	32.0
IMRT	26.7	22.6	60.6
Unknown	3.5	0.0	7.4
Totals	100.0	100.0	100.0

(*) weighted%.

*EBRT*, external beam radiotherapy; *IMRT*, intensity modulated radiation treatment.

The percent of patients receiving different levels of radiation dose varied among regions and states. The proportion of men receiving the high dose (≥75 Gy) varied from a high of 41% in Region E to a low of 14% in Region C (p < 0.0001). The dose received was not related to marital status or proportion of working class residents in the patient’s census tract; however, those in areas with population indicators reflecting a higher socioeconomic status (i.e., higher proportion of persons with at least a high school degree and lower proportion living under the poverty level) as well as those living in an urbanized area were more likely to receive a high dose of ≥75 Gy (p < 0.03). Clinical T stage and ACE-27 comorbidity score were not associated with level of total EBRT dose administered; however, patients with high pre-treatment PSA of >20 were less likely to receive high dose EBRT than their counterparts with lower pre-treatment PSA of ≤ 20 (p = 0.0016). Higher Gleason score was associated with a higher percentage of low dose EBRT and less medium dose EBRT (70-74 Gy) [p = 0.0007]. The percentages of high dose EBRT were similar across the Gleason score groups.

Total dose received also varied significantly across the recurrence risk groups (p = 0.0001) (Table 3). Patients in the HR group were the most likely (61.8%) to receive the lowest dose (<70 Gy), compared to 53.8% and 44.3% of those with IR and LR, respectively. Intermediate dose (70-74 Gy) was more often used for LR patients, while the high dose (≥75 Gy) radiation was more often used for IR patients.

## Discussion

The association of PC treatment with clinical factors such as PSA, GS and clinical T stage or modified risk groups has been reported previously for PC cases diagnosed in 1997 [3], 1999 [13], 2002 [14], 2013 [15]. For purposes of PC management, however, it is important to examine patterns of care according to the NCCN recurrence risk groups, which already take into account the three most important predictors of prognosis (i.e., clinical T stage, pretreatment PSA and biopsy GS). Although each of these factors independently predicts relapse-free survival, multiple studies have demonstrated that the recurrence risk groups, representing a combination of factors, better categorize patients with different prognosis in order to reliably compare the success of treatment regimens [1]. The current study examines the initial RT patterns in a large number of study subjects with initial RT details (dose and technique) by the NCCN recurrence risk group.

The results of this study reflect the major changes in the radiotherapeutic management of localized PC in the United States that have occurred. RT planning and delivery systems have become more sophisticated and precise, and have moved from conventional RT to IMRT. IMRT is able to deliver a much higher conformal dose to tumor targets than conventional RT through modulation of radiation beam intensities in many different fields [16]. High-dose RT is known to improve disease control in PC, particularly with intermediate risk factors [17]. In this study based on cases diagnosed in 2004, high-dose RT (≥75 Gy EBRT or combination of EBRT and seed implant BT) was used more often in the IR group than in the LR or HR groups (Figure 1). IMRT was often utilized to deliver high dose in patients receiving EBRT only. Use of high-dose RT for patients in the IR group with the growing popularity of IMRT has not been reported in previous patterns of care studies [3,13-15]. IMRT was not commonly used until the first decade of this century, and therefore might not be a variable to be abstracted for analysis in previous studies.

Long-term results of various randomized trials from the cooperative groups including the Radiation Therapy Oncology Group (RTOG) and the European Organization for the Research and Treatment of Cancer have indicated clear advantages of combining RT with HT for patients with high-risk localized PC [18,19]. In addition, results of a randomized study [20] showed that addition of short-term (6-month) HT to EBRT (70 Gy in 35 daily fractions) conferred an overall survival benefit (88% for EBRT plus HT versus 57% for RT alone) after a median follow-up of 4.52 years in 206 non-metastatic PC patients (majority in the IR group). It appears that the results from these randomized trials have influenced routine management of PC in the community. In our study, patients in IR or HR were more likely to receive RT plus HT than LR patients in this study, even though use of RT combined with HT was not recommended to treat patients in IR in the 2002 NCCN guidelines. It is worth noting that the role of HT added to high-dose RT in IR still remains unclear. RTOG is currently investigating the addition of short-term HT to high-dose RT to improve survival in patients with intermediate risk factors.

One of the limitations of our study is the possible underreporting of radiation and hormone treatment derived from the registry data or data re-abstraction. In addition, use of life expectancy as a key decision making factor in determining the NCCN guideline care could not be addressed in our analyses.

## Conclusion

This is the largest patterns of care study to describe specific types and doses of radiotherapy used to treat prostate cancer patients in different NCCN recurrence risk groups using state registry data. We found the use of seed implant brachytherapy to be more common in PC patients with low risk factors compared to patients in the intermediate to high-risk groups, while radiotherapy combined with hormone therapy was used more often in patients with higher risk factors than in patients with low risk group. High dose RT (≥75 Gy EBRT or EBRT combined with seed implant brachytherapy) was more often given to patients in the intermediate risk group than to low-risk or high-risk patients. IMRT has been increasingly accepted as the radiotherapy of choice and is often used to deliver high-dose EBRT. The results of this study provide a basis for assessing the use of radiation therapy and for monitoring trends in its delivery.

## Abbreviations

PC: Prostate cancer; IMRT: Intensity modulated radiation treatment; LR: Low risk; IR: Intermediate risk; HR: High risk; NCCN: National comprehensive cancer network; HT: Hormone therapy; BT: Brachytherapy; EBRT: External beam radiotherapy; NPCR: National program of cancer registries; CDC: Center for disease control and prevention; POC BP: Prostate cancer data quality and patterns of care study; PSA: Prostate specific antigen; GS: Gleason score; ACE-27: Adult comorbidity evaluation-27; Gray: Gy; CI: Confidence intervals; OR: Odds ratios.

## Competing interests

The authors declare that they have no competing interests.

## Authors’ contributions

DW carried out the design of the study, participated in data analysis and drafted manuscript. AH participated in the design of the study, and helped with data analysis. ASH participated in the design of the study, contributed to the data collection, and revised the manuscript critically for important intellectual content. XCW participated in the design of this study, contributed to the data collection and revised the manuscript critically for important intellectual content. ML participated in the design of the study and helped with the data analysis. SF participated in the design of the study, contributed to the data collection, and revised the manuscript critically for important intellectual content. MG participated in the study design, contributed to the data collection and revised manuscript critically for important intellectual content. TT participated in the design and coordination of the study, and revised the manuscript critically for important intellectual content. JO participated in the design and coordination of the study, and helped draft the manuscript and revised the manuscript critically for important intellectual content. All authors read and approved the final manuscript.
